# Central glutathione mitigates obesity-induced social phobia: evidence from human and animal studies

**DOI:** 10.20945/2359-4292-2025-0016

**Published:** 2025-08-25

**Authors:** Yurui He, Kai Xiang, Yanli Chen, Hongfang Zheng, Youde Liu, Jingjing Li, Yan Zhang, Rui Jiang, Jinping Sheng

**Affiliations:** 1 Department of Radiology, The General Hospital of Western Theater Command, Chengdu, 610083, Sichuan Province, China

**Keywords:** Glutathione, Obesity, Oxidative stress, Phobia, social

## Abstract

**Objective:**

To explore the impact of obesity on social phobia and the therapeutic effect of the antioxidant glutathione on this disorder.

**Methods:**

A total of 600 subjects were divided into an Obese Group and a Control Group. Four scales were utilized to determine social phobia, and magnetic resonance spectroscopy was applied to evaluate the central glutathione levels. Multivariate logistic regression was used for analysis. A mouse obesity model was subsequently established using a high-fat diet. Agonists or inhibitors were used to upregulate or downregulate hippocampal glutathione. The social interaction test was used to evaluate the social phobia of the mice, and the ELISA method was used to measure the levels of oxidative stress indicators in the hippocampus. Anova was used for analysis.

**Results:**

Compared with the control subjects, obese subjects had higher social phobia scores. A higher central glutathione level was associated with a lower social phobia score. In the animal experiments, obese mice exhibited more social phobia behaviours. When the glutathione level in the hippocampus was increased and decreased, the oxidative stress level in the hippocampus of the mice decreased and increased accordingly, whereas social phobia behaviours were alleviated and exacerbated, respectively.

**Conclusion:**

Obesity may induce social phobia. However, the antioxidant glutathione attenuates the central oxidative stress response and alleviates obesity-related social phobia.

## INTRODUCTION

Many studies have confirmed that obesity can impair cognitive function and increase the risk of psychological disorders such as anxiety and depression (^1^,^2^,^3^,^4^,^5^,^6^). However, the underlying mechanisms remain unclear, yet oxidative stress clearly plays a key role (^7^,^8^,^9^). Typically, obesity may disrupt the body's microenvironment, trigger low-grade chronic inflammation, damage mitochondrial function, and lead to systemic oxidative stress. Excessive oxidative stress subsequently suppresses the endogenous antioxidant system, intensifies neuroinflammation, changes the anatomical structure of the brain, and ultimately damages normal brain functions, especially those that involve cognitive and psychological regulation.

Social phobia, also known as social anxiety disorder, is a relatively common type of psychological disorder that can present symptoms in three aspects: emotional, behavioural, and cognitive (^10^). However, to date, there has been no research specifically focused on the correlation between obesity and the risk of social phobia.

Glutathione is an important endogenous antioxidant that can scavenge reactive oxygen species by donating electrons, reduce oxidative damage, and play a vital role in maintaining the intracellular redox balance (^11^). In the brain, glutathione is widely distributed and exerts significant neuroprotective effects, which include improving cognitive function (^12^). A series of animal experiments have also indirectly suggested the influence of glutathione on anxiety. In brief, inhibiting 2-oxoglutarate dehydrogenase in the rat brain can reduce the redox state of glutathione and exacerbate behaviours indicative of anxiety (^13^).

Intermittent hypoxia can increase the expression of glutathione reductase 1 in the mouse brain and alleviate anxiety-related behaviours (^14^). Additionally, knocking out glutathione peroxidase 4 in the dopaminergic neurons of the ventral midbrain of mice can also trigger behaviours characteristic of anxiety (^15^). Therefore, we hypothesized that glutathione also has a potentially positive effect on social phobia, although this hypothesis has not yet been proven.

On the basis of the abovementioned background, this study planned to recruit hundreds of obese subjects to determine the correlations among obesity, central/peripheral glutathione levels, and the risk of social phobia. Subsequently, an obese mouse model was established, and its glutathione levels were increased and suppressed to verify the impact of obesity on this psychological disorder as well as the protective effect of glutathione against it. Through this research, we hoped to gain a deeper understanding of the pathogenesis and treatment methods of social phobia.

The objective od this study was to explore the impact of obesity on social phobia and the therapeutic effect of the antioxidant glutathione on this disorder.

## MATERIALS AND METHODS

### Case-control study

First, 300 patients who underwent imaging examinations at The General Hospital of Western Theater Command from January 1, 2020, to February 17, 2024, were consecutively enrolled. The inclusion criteria were as follows: aged 18 years or older; Asian; more than 9 years of education; body mass index (BMI) of 23 kg/m^2^ or more according to the Asian standard for overweight and obesity of the World Health Organization (WHO) (^16^); no acute or serious diseases such as acute infection, acute myocardial infarction, or malignant tumour; no prior diagnosis of cognitive or psychological diseases; no intake of oral antioxidants such as glutathione, vitamin C, and vitamin E in the past 12 months; and understanding the study process and consenting to participate. Those not meeting these criteria were excluded. The selected subjects formed the obesity group.

For each subject included in the obesity group, on the same day of their inclusion or the subsequent day, one additional subject was randomly selected from the eligible normal-weight candidates who were undergoing imaging examinations. These 300 selected subjects formed a Control Group. The inclusion criteria for the Control Group were as follows: BMI lower than 23 kg/m2 but not less than 18.5 kg/m2 (^16^); sex and age of the subjects in the Control Group were matched one-to-one with those of the subjects in the obesity group; and all other inclusion criteria were the same as those of the obesity group.

Second, data on the characteristics of all the subjects, including sex, age, ethnicity, smoking history, drinking history, peripheral serological indicators, history of chronic diseases, and relevant medication history, were collected by reviewing medical records. Here, a smoking history was defined as continuous or cumulative regular smoking for at least 12 months within the past 10 years; a drinking history was defined as continuous or cumulative regular alcohol consumption for at least 12 months within the past 10 years; a history of various chronic diseases was determined according to the diagnostic certificates in medical records; and a medication history was defined as having regularly taken relevant medications in the last 12 months.

Third, the social phobia of each subject was evaluated using four scales:

- The Liebowitz Social Anxiety Scale (LSAS) helps diagnose social phobia and measure its severity by quantifying fear and avoidance behaviours in social situations (^17^). The total score is 144 points. A score exceeding 60 points may suggest the presence of social phobia. The total scores for mild, moderate, and severe social phobia are 60 to 74 points, 75 to 94 points, and 95 points or above, respectively.
- The Social Avoidance and Distress Scale (SADS) is mainly used to evaluate an individual's tendency to avoid social situations and the degree of distress resulting from them (^18^). The total score is 112 points. A score exceeding 60 points is considered to indicate relatively serious problems in social avoidance and distress, suggesting social phobia.
- The Social Anxiety Scale (SAS) is used to measure the anxiety that individuals experience in social situations (^19^). The total score is 80 points, and a score exceeding 50 points indicates the presence of a tendency towards social anxiety.
- The Brief Social Phobia Scale (BSPS) is used to assess the severity of an individual's social phobia symptoms quickly and concisely (^20^). The total score is 30 points, and a score exceeding 15 points indicates the presence of a tendency towards social phobia. Fourth, a 5-mL whole-blood sample was collected from each subject. Plasma samples were obtained by centrifugation and stored at -80 °C. Plasma glutathione levels were determined using Tietze's modified method (^21^). The assay kit was purchased from Cayman, USA (#703002).

Fifth, the central glutathione level of each subject was determined by magnetic resonance spectroscopy, and the measurement results were quantified into absolute concentration values (millimoles) using an external reference (^22^,^23^). For specific details, please refer to Supplemental Table 1.

Sixth, the statistical analysis strategies used are as follows. Continuous variables are expressed as the means ± standard deviations, and the differences between groups were evaluated using the independent samples *t* test. Categorical variables are expressed as frequencies (composition ratios), and the differences between groups were evaluated using the Chi-squared test. The linear relationship between two continuous variables was assessed using Pearson's linear correlation. The multivariate correlation between two variables was assessed using multivariate logistic regression. In this analysis, a series of characteristics of the subjects were adjusted. A p-value < 0.05 indicated that the difference or correlation was statistically significant.

### Animal experiments

First, 50 C57BL/6J mice (aged 6 to 12 weeks, males/ females=1:1) were purchased from the Laboratory Animal Center of Sichuan University. They were housed in a comfortable and quiet environment for one week and provided with clean drinking water, nutritionally balanced feed and a 24-hour cycle of day-night alternation. The mice were then randomly divided into five groups, namely, the Control Group, the obesity group, the placebo group, the agonist group, and the inhibitor group, with ten mice in each group.

Second, starting from the second week, except for the mice in the Control Group, the mice in the other four groups were fed a high-fat diet (with 60% of the energy coming from fats, 20% from carbohydrates, and 20% from proteins) for 16 weeks (D12492, Research Diets). Moreover, the mice in the Control Group were fed a normolipidic diet (D12450B, Research Diets) during this period. Afterwards, the body weights of the male mice on a high-fat diet all reached between 30 and 35 g, whereas those of the female mice all reached between 26 and 30 g, with obvious fat accumulation in their abdomens. In contrast, the body weights of the male and female mice in the Control Group were between 24 and 29 g and between 18 and 22 g, respectively. These results indicated that the obesity model was successfully validated.

Third, after the model was established, a 2-week intervention was carried out on the mice. In the agonist group, each mouse was given N-acetylcysteine (NAC) dissolved in normal saline and administered via gavage needle once a day at a dose of 300 mg/kg for a total of 2 weeks (^24^). In the inhibitor group, each mouse was given buthionine sulfoximine (BSO) dissolved in normal saline and administered via gavage with a gavage needle once every other day at a dose of 3 mmol/kg for a total of two weeks (^25^). In the placebo group, each mouse was given normal saline and administered via gavage with a gavage needle once every other day at a dose of 1 mL for a total of 2 weeks. The remaining two groups did not receive any intervention measures.

Fourth, after the intervention, all the mice were evaluated for social phobia using the social interaction test (^26^). The equipment and specific procedures are listed in Supplemental Table 2. The behavioural performance of the mice within 10 minutes was subsequently reported, including the percentage of dwelling time in the social area where the unfamiliar mouse was present (dwelling time), the dwelling frequency of entering that social area (dwelling frequency), and the interaction count of direct interactions with the unfamiliar mouse (interaction count).

Fifth, all the mice were euthanized via exsanguination after anaesthesia. The skull was subsequently opened using a conventional method. Hippocampal tissue was dissected on ice and rinsed with prechilled phosphate-buffered saline. Then, the hippocampal tissue was minced, and tissue lysis buffer containing protease inhibitors was added. The tissue was thoroughly homogenized with a homogenizer in an ice bath. Finally, the homogenate was transferred to a centrifuge tube and centrifuged at a speed of 10,000 rpm in a low-temperature centrifuge for 15 minutes. The supernatant was extracted and stored in a -80 °C freezer for subsequent testing. The levels of glutathione, malondialdehyde (MDA), 8-hydroxydeoxy-guanosine (8-OHdG), protein carbonyl derivative (PCD), interleukin 6 (IL-6), and tumour necrosis factor-alpha (TNF**-**α) in the homogenate were detected by ELISA. The commercial kits used are listed in Supplemental Table 3. All operations were carried out in strict accordance with the manufacturer's instructions.

Sixth, the statistical analysis strategies used are as follows. Continuous variables are expressed as the means ± standard deviations, and the differences between groups were evaluated using the analysis of variance (Anova) and least significant difference (LSD) analysis. A p-value < 0.05 indicated that the difference was statistically significant.

## RESULTS

### Case-control study

As shown in Table 1, the BMIs of the obese and Control Groups were 25.6 ± 1.4 kg/m^2^ and 20.9 ± 1.2 kg/m^2^, respectively. Compared with the Control Group, the Obese Group had a greater prevalence of smoking history and a history of hypertension or stroke, higher peripheral levels of triglycerides and total cholesterol, and lower peripheral levels of albumin (p < 0.05). Additionally, compared with those in the Control Group, more subjects in the Obese Group took calcium antagonists, angiotensin-converting enzyme inhibitors/angiotensin II receptor antagonists, or statins (p < 0.05). These findings suggest that the majority of the characteristics of the subjects were balanced between the two groups, while several characteristics presented statistically significant differences between them.

**Table 1:** Characteristics of subjects in the Obese Group *versus* the Control Group

Variable	Obese group (n = 300)	Control group (n = 300)	t/ χ^2^ value	p-value
Male	172 (57.3)	156 (52.0)	1.722	0.189
Age, years	65.7 ± 7.0	64.8 ± 7.2	1.545	0.123
HAN	273 (91.0)	285 (95.0)	3.687	0.055
Smoking	69 (23.0)	44 (14.7)	6.814	0.009
Drinking	55 (18.3)	42 (14.0)	2.078	0.149
BMI, kg/m^2^	25.6 ± 1.4	20.9 ± 1.2	43.564	< 0.001
TG, mmol/L	2.2 ± 0.7	1.5 ± 0.6	13.252	< 0.001
TC, mmol/L	5.5 ± 0.9	4.0 ± 0.9	19.725	< 0.001
ALB, g/L	39.2 ± 2.9	39.8 ± 3.1	2.828	0.005
UA, µmol/L	257.6 ± 86.1	268.1 ± 84.6	1.504	0.133
TBIL, µmol/L	10.6 ± 3.7	10.9 ± 3.8	0.965	0.335
T2DM	63 (21.0)	50 (16.7)	1.843	0.175
CHD	52 (17.3)	39 (13.0)	2.189	0.139
HTN	128 (42.7)	87 (29.0)	12.185	< 0.001
Stroke	36 (12.0)	20 (6.7)	5.042	0.025
CCB	88 (29.3)	56 (18.7)	9.357	0.002
ACEI/ARB	65 (21.7)	42 (14.0)	6.017	0.014
β-blocker	36 (12.0)	27 (9.0)	1.437	0.231
Nitrates	41 (13.7)	32 (10.7)	1.263	0.261
Statins	55 (18.3)	30 (10.0)	8.567	0.003
Biguanides	32 (10.7)	27 (9.0)	0.470	0.493
Sulfonylureas	30 (10.0)	24 (8.0)	0.733	0.392
AGI	19 (6.3)	17 (5.7)	0.118	0.731

Results expressed as n (%) or mean ± standard deviation. Differences between groups were evaluated by independent samples *t* test. Categorical variables were expressed by frequency (composition ratio), and differences between groups were evaluated by Chi-squared test. p < 0.05 indicated that the differences were statistically significant.

HAN: Han Chinese; BMI: body mass index; TG: triglycerides; TC: total cholesterol; ALB: albumin; UA: blood uric acid; TBIL: total bilirubin; T2DM: type 2 diabetes mellitus; CHD: coronary heart disease; HTN: hypertension; CCB: calcium antagonists; ACEI/ARB: angiotensin-converting enzyme inhibitors/ angiotensin II receptor antagonists; AGI: alpha glycosidase inhibitors.

As shown in Table 2, both the central and peripheral glutathione levels were lower in the Obese Group than in the Control Group (p < 0.001; p < 0.001). The scale scores of the LSAS, SADS, SAS and BSPS were higher in the Obese Group than in the Control Group (p < 0.001; p < 0.001; p < 0.001; p < 0.001). In addition, according to the LSAS, there were more patients with social phobia in the Obese Group than in the Control Group (p < 0.001), and there were also more patients with moderate-severe social phobia in the Obese Group than in the Control Group (p = 0.008). These findings show that the subjects in the Obese Group have lower levels of glutathione in their bodies and that their levels of social phobia are relatively greater.

**Table 2:** Glutathione levels and social phobia scores of subjects in the obesity group and the Control Group

Variable	Obese group (n = 300)	Control group (n = 300)	t/ χ^2^ value	p-value
Glutathione,mmol/L				
Peripheral level	0.65 ± 0.14	0.82 ± 0.20	12.090	< 0.001
Central level	0.96 ± 0.21	1.20 ± 0.23	13.445	< 0.001
Scale score, unitless				
LSAS	56.1 ± 10.9	46.0 ± 11.5	10.961	< 0.001
SADS	57.1 ± 8.4	49.4 ± 8.7	11.025	< 0.001
SAS	47.5 ± 6.1	42.4 ± 6.6	9.857	< 0.001
BSPS	14.2 ± 2.7	12.3 ± 2.7	8.520	< 0.001
LSAS, social phobia				
Presence	84 (28.0)	28 (9.3)	34.426	< 0.001
Absent	216 (72.0)	272 (90.7)		
LSAS, degree				
Mild	61 (20.3)	27 (9.0)	7.071	0.008
Moderate - severe	23 (7.7)	1 (0.3)		

Results expressed as mean ± standard deviation. Differences between groups were evaluated by independent samples *t* test. Categorical variables were expressed by frequency (composition ratio), and differences between groups were evaluated by Chi-squared test. p < 0.05 indicated that the differences were statistically significant.

LSAS: Liebowitz Social Anxiety Scale; SADS: Social Avoidance and Distress Scale; SAS: Social Anxiety Scale; BSPS: Brief Social Phobia Scale.

As shown in Table 3, after adjusting for a series of confounding factors, the multivariate model suggested that obesity was associated with lower levels of peripheral glutathione and central glutathione and higher LSAS, SADS, SAS and BSPS scores (p < 0.001). These findings suggest that there is an independent correlation between obesity and the level of glutathione in the body and that there is also an independent correlation between obesity and the degree of social phobia of the subjects.

**Table 3:** Multivariate relationship between obesity and glutathione levels/ social phobia scores of subjects in the study

Variable	Univariate OR (95%CI)	p-value
Obesity		
Peripheral glutathione	0.231 (0.166 ~ 0.321)	< 0.001
Central glutathione	0.178 (0.121 ~ 0.263)	< 0.001
LSAS score	3.778 (2.377 ~ 6.005)	< 0.001
SADS score	3.602 (2.303 ~ 5.633)	< 0.001
SAS score	3.832 (2.379 ~ 6.173)	< 0.001
BSPS score	3.093 (2.018 ~ 4.742)	< 0.001

Variable	Multivariate OR (95%CI)	p-value
Obesity		
Peripheral glutathione	0.229 (0.165 ~ 0.319)	< 0.001
Central glutathione	0.179 (0.121 ~ 0.264)	< 0.001
LSAS score	3.896 (2.443 ~ 6.211)	< 0.001
SADS score	3.714 (2.368 ~ 5.826)	< 0.001
SAS score	3.885 (2.408 ~ 6.268)	< 0.001
BSPS score	3.166 (2.060 ~ 4.863)	< 0.001

Multivariate logistic regression was adopted to assess the relationship between glutathione levels and social phobia scores. Multivariate model was adjusted for a series of characteristics of subjects. p < 0.05 suggested a statistically significant correlation.

OR: odds ratio; 95%CI: 95% confidence interval; LSAS: Liebowitz Social Anxiety Scale; SADS: Social Avoidance and Distress Scale; SAS: Social Anxiety Scale; BSPS: Brief Social Phobia Scale,

As shown in Table 4, both the peripheral and central levels of glutathione were negatively linearly associated with the LSAS, SADS, SAS and BSPS scores (p < 0.001). As shown in Table 5, after adjusting for a series of confounding factors, multivariate logistic regression suggested that higher levels of central glutathione were significantly associated with decreased LSAS, SADS, SAS and BSPS scores (p < 0.001; p < 0.001; p < 0.001; p < 0.001, respectively). Multivariate logistic regression also suggested that higher levels of peripheral glutathione were significantly associated with decreased LSAS and SAS scores (p = 0.009; p = 0.049) but not with SADS or BSPS scores (p = 0.058, p = 0.061). In addition, the results of the univariate logistic regression are listed in Supplemental Table 4. The above findings indicate that the levels of glutathione in the body (especially central glutathione) are significantly correlated with the levels of social phobia.

**Table 4:** Linear relationship between glutathione levels and social phobia scores of subjects in the study

Variable/ glutathione	Variable/ social phobia	r value	p-value
Peripheral level	LSAS score	-0.185	< 0.001
Peripheral level	SADS score	-0.172	< 0.001
Peripheral level	SAS score	-0.175	< 0.001
Peripheral level	BSPS score	-0.174	< 0.001
Central level	LSAS score	-0.402	< 0.001
Central level	SADS score	-0.392	< 0.001
Central level	SAS score	-0.382	< 0.001
Central level	BSPS score	-0.345	< 0.001

LSAS: Liebowitz Social Anxiety Scale; SADS: Social Avoidance and Distress Scale; SAS: Social Anxiety Scale; BSPS: Brief Social Phobia Scale.

Pearson's linear regression was adopted to assess the relationship between glutathione levels and social phobia scores. p < 0.05 suggested a statistically significant correlation.

**Table 5:** Multivariate relationship between glutathione levels and social phobia scores of subjects in the study

Variable	Model	OR (95%CI)	p-value
Peripheral glutathione			
LSAS score	Multivariate	0.629 (0.445 ~ 0.889)	0.009
SADS score	Multivariate	0.724 (0.518 ~ 1.011)	0.058
SAS score	Multivariate	0.704 (0.496 ~ 0.999)	0.049
BSPS score	Multivariate	0.731 (0.526 ~ 1.014)	0.061
Central glutathione			
LSAS score	Multivariate	0.384 (0.260 ~ 0.568)	< 0.001
SADS score	Multivariate	0.389 (0.265 ~ 0.570)	< 0.001
SAS score	Multivariate	0.342 (0.229 ~ 0.512)	< 0.001
BSPS score	Multivariate	0.467 (0.322 ~ 0.676)	< 0.001

Multivariate logistic regression was adopted to assess the relationship between glutathione levels and social phobia scores. Multivariate model was adjusted for a series of characteristics of subjects. p < 0.05 suggested a statistically significant correlation.

OR: odds ratio; 95%CI: 95% confidence interval; LSAS: Liebowitz Social Anxiety Scale; SADS: Social Avoidance and Distress Scale; SAS: Social Anxiety Scale; BSPS: Brief Social Phobia Scale.

### Animal experiments

**Figure 1:**
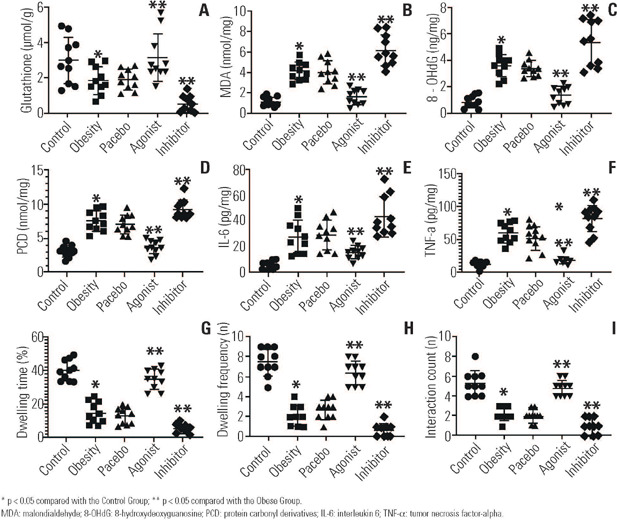
The results of animal experiments.

As shown in Figure 1A, compared with that in the Control Group, the level of glutathione in the hippocampus of the Obese Group was significantly lower (p < 0.05). Compared with that in the Obese Group, the level of glutathione in the hippocampus of the mice in the agonist group was significantly greater (p < 0.05), whereas the level of glutathione in the hippocampus of the mice in the inhibitor group was significantly lower (p < 0.05). These results confirmed the impact of obesity on the level of glutathione in the hippocampus, as well as the effectiveness of related agonists and inhibitors.

* p < 0.05 compared with the Control Group; ** p < 0.05 compared with the Obese Group.

MDA: malondialdehyde; 8-OHdG: 8-hydroxydeoxyguanosine; PCD: protein carbonyl derivatives; IL-6: interleukin 6; TNF-α tumor necrosis factor-alpha.

As shown in Figures 1B, 1C, 1D, 1E, 1F, compared with those in the Control Group, the levels of MDA, 8-OHdG, PCD, IL-6 and TNF-α in the hippocampi of the mice in the obesity group were significantly greater (p < 0.05). Compared with those in the Obese Group, the levels of the above oxidative stress and inflammation indicators in the hippocampi of the mice in the agonist group were significantly lower (p < 0.05), whereas the levels of these indicators in the hippocampi of the mice in the inhibitor group were significantly greater (p < 0.05). These results confirmed that obesity can lead to an increase in the levels of oxidative stress and inflammation in the hippocampus and that glutathione can alleviate this situation.

As shown in Figure 1G, 1H, 1I, compared with those in the Control Group, the dwelling times of the mice in the obesity group in the social interaction test were shorter (p < 0.05), and the dwelling frequency and interaction count were lower (p < 0.05). Compared with those in the obesity group, these indicators in the agonist group were longer and greater (p < 0.05), whereas the indicators in the inhibitor group were shorter and lower (p < 0.05). These results confirmed that obesity led to social phobia behaviours in mice and that glutathione played a protective role in this regard.

## DISCUSSION

Currently, obesity ranks among the top ten chronic diseases of humans listed by the WHO. In 2020, 2.2 billion adults worldwide were overweight or obese, and this number is projected to reach 3.3 billion by 2035 (^27^). Obesity not only impairs physical health but also increases the risk of psychological disorders. For example, previous studies have indicated that obesity is a significant risk factor for anxiety and depression (^5^,^6^). Therefore, this study utilized a case-control study and animal experiments to verify the correlation between obesity and social phobia. Considering the harmful effects of oxidative stress in obesity-related diseases, this study also explored the protective effect of the antioxidant glutathione on obesity-related social phobia at these two levels.

In the case-control study, both univariate and multivariate analyses revealed that obesity was significantly associated with increased scores on the four social phobia scales. Considering the subjectivity of the scales, this study used four scales simultaneously, and the consistent results increased the stability of the findings. However, these results cannot rule out the possibility that social phobia, along with the consequent lack of exercise, led to obesity. Therefore, the subsequent animal experiments revealed that, compared with control mice, obese mice with successfully established models presented manifestations of social phobia. Collectively, the above results confirmed that obesity might be a potential risk factor for social phobia.

The study also revealed that, compared with those in the Control Group, the glutathione levels in both the central and peripheral areas of obese subjects were significantly lower. Since glutathione is a key antioxidant, the reduction in its level could be attributed to increased oxidative stress. In the animal experiments, obese mice presented a decrease in glutathione levels and an increase in the levels of these two oxidants, indicating an excessive oxidative stress response. Many previous studies have confirmed that obesity can trigger systemic oxidative stress (^28^). These findings constitute the basis for glutathione exerting a protective effect against social phobia.

Finally, through Pearson linear correlation and multivariate logistic regression, this study confirmed a significant correlation between an increase in central glutathione levels and a decrease in the scores of the four social phobia scales. The results of the four scales were consistent, which also enhanced the stability of the findings. In the animal experiment section, by increasing and decreasing glutathione levels in the hippocampal tissue of mice, oxidative stress and inflammatory responses were inhibited and activated, respectively, thus improving and worsening social phobia. These results suggest that central glutathione could effectively inhibit oxidative stress and improve social phobia caused by obesity.

Notably, this study also confirmed that an increase in the peripheral glutathione level was significantly correlated with a decrease in the scores of only two social phobia scales and was not related to the scores of the other two scales. Therefore, the evidence for the correlation between peripheral glutathione and social phobia is less sufficient than that for the correlation between central glutathione and social phobia. Sample differences may be a potential explanation for these results, which requires repeated verification through more studies. Another potential pathophysiological explanation involves the role of the blood-brain barrier. The blood-brain barrier is a highly specialized structure composed of brain capillary endothelial cells, basement membranes, and the endfeet of astrocytes, among other components. Endothelial cells are tightly connected, forming a barrier that restricts the free diffusion of substances. Only specific small molecules and some substances produced by special transport proteins or receptors can enter the brain through the blood-brain barrier. Previous animal experiments have shown that although glutathione can cross the blood-brain barrier, the transport mechanism it relies on has a low affinity, is saturable, and is easily affected by factors such as age (^29^,^30^). Therefore, the quantity and efficiency of its entry into and exit from the central nervous system are restricted, resulting in differences in glutathione levels between the peripheral and central nervous systems. Peripheral glutathione cannot directly affect the brain regions related to social phobia, thus influencing its correlation with social phobia.

The main innovations of this study are as follows: First, this is the first in-depth exploration of the relationship between the level of glutathione in the body (a key indicator of oxidative stress) and social phobia. Second, systematic analyses are carried out at two levels, namely, observational studies and animal experiments. These two approaches mutually confirm each other, providing more solid evidence. Third, new ideas are proposed for the intervention and treatment of obesity-related social phobia.

This study failed to comprehensively reveal the mechanism by which glutathione relieves obesity-related social phobia, which may be a potential limitation of this study. However, on the basis of existing medical knowledge, we can speculate the following possible mechanisms: first, as an important antioxidant in the human body, glutathione can scavenge excess free radicals in the body and participate in maintaining the activity of the antioxidant enzyme system (such as glutathione peroxidase and glutathione S-transferase), thereby alleviating the damage to the central nervous system caused by oxidative stress. Second, glutathione can inhibit the neuroinflammatory response and restore the normal microenvironment of the central nervous system by reducing the production and release of proinflammatory cytokines and decreasing the excessive activation of microglia (^31^,^32^). Third, glutathione may affect the functions of γ-aminobutyric acidergic neurons and glutamatergic neurons, regulate the metabolism of γ-aminobutyric acid and glutamate, stabilize the concentrations of these two neurotransmitters in the central nervous system, and maintain the balance of neural signal transmission (^33^,^34^). However, the above speculations still need to be verified by future studies.

In addition to the failure to explore in depth the mechanism by which glutathione alleviates obesity-related social phobia, other limitations of this study are as follows: the cross-sectional design cannot rule out the potential reverse effect of social phobia on the risk of obesity; dose-response relationships between glutathione and related signalling pathways have not been revealed; this study explored only the phenotypic level and did not conduct further research at the genetic level. These limitations are due to the lack of objective scientific research conditions. To address the above limitations, future research can focus on three aspects. Epidemiologically, conduct large-scale longitudinal cohort studies to clarify the bidirectional relationship between obesity and social phobia, as well as the dose-response relationship between glutathione and obesity-related social phobia. At the cellular and molecular levels, proteomics and metabolomics have been used to explore signalling pathway mechanisms. Genetically, genome-wide association studies and gene editing technologies have been leveraged to identify regulatory genes and drug targets.

In conclusion, this study confirmed that obesity can induce social phobia and identified the oxidative stress response as a crucial link in the onset and progression of obesity-related social phobia. This study further revealed that the antioxidant glutathione can play a key therapeutic role in the central nervous system, providing a new target for improving obesity-related social phobia. This discovery not only renovates the academic community's understanding of the pathogenesis of social phobia but also opens a new path for the innovation of clinical intervention strategies. In the future, intervention methods such as supplementation with NAC to increase the level of glutathione are expected to be translated into practical and effective treatment plans, opening a new direction for the clinical treatment of obesity-related social phobia.

## SUPPLEMENTAL MATERIAL

**Supplementary Table 1:** Detection of central glutathione in subjects

Item	Description
Brief introduction	Nuclear magnetic resonance spectroscopy is an analytical technique based on the magnetism of atomic nuclei. It can utilize the characteristics of magnetic atomic nuclei (such as hydrogen nuclei, carbon nuclei etc.) to absorb and emit radiofrequency radiation in a magnetic field to obtain information about molecular structures and dynamic behaviors
Detailed process	The following is a further refinement of the steps for detecting the central glutathione level in patients using nuclear magnetic resonance spectroscopy: 1. Instrument and preparation: Use the Achieva 3.0T superconducting nuclear magnetic resonance spectroscopy analyzer produced by Marconi, USA, to carry out the detection work. Before the formal detection, a comprehensive calibration and debugging of the instrument are required to ensure that all performance indicators of the instrument meet the detection requirements, such as the uniformity of the magnetic field strength and the accuracy of the radiofrequency pulse. At the same time, prepare the external reference for quantification - the glutathione model, and check its concentration accuracy and stability to ensure the reliability of subsequent concentration calculations 2. Image acquisition and region of interest selection: - First, perform routine axial magnetic resonance image acquisition on the patient to obtain clear brain images, providing a basis for the subsequent selection of the region of interest - In the bilateral proximal mesial temporal lobe regions, based on the knowledge of anatomical structures and image features, accurately select a cubic volume of interest with a size of 1 cm x 1 cm x 1 cm. During the selection process, with the aid of the auxiliary tools of the image analysis software, carefully observe and avoid tissues such as the apical fat of the petrous bone, peripheral large blood vessels, and cerebrospinal fluid in the sulci. To ensure the accuracy of the selection, at least two experienced radiologists can jointly confirm the location and scope of the region of interest 3. Shimming of the region of interest: - Carry out the shimming operation on the selected region of interest with the aim of making the magnetic field within this region more uniform, thereby improving the quality of the spectrum - Adopt a specialized shimming program and algorithm. By adjusting the current of the shimming coils of the instrument, continuously optimize the uniformity of the magnetic field. During this process, monitor the full width at half maximum value of the water peak in real time, and keep adjusting until the full width at half maximum of the water peak is less than 8 Hz, reaching the required spectral resolution standard of 0.1 ppm. After each adjustment, wait for a period of time for the magnetic field to stabilize before conducting the next measurement and evaluation to ensure the accuracy of the measurement results 4. Measurement of the water peak frequency and water suppression: - Precisely measure the frequency of the water peak within the region of interest, using a high-precision frequency measurement module and algorithm to obtain accurate water peak frequency data - Implement the water suppression operation using a chemical shift-selective excitation sequence. This sequence excites water molecules through a radiofrequency pulse of a specific frequency, suppressing their signals. During the operation, monitor the ratio of the height of the water peak to that of other metabolites in real time, and continuously adjust the excitation parameters, such as the intensity, frequency, and duration of the radiofrequency pulse, until the ratio of the water peak to the peak height of other metabolites is less than 100, ensuring that the water signal will not interfere with the glutathione signal 5. Spectrum acquisition: - Use the stimulated-echo acquisition mode sequence for spectrum acquisition. Strictly set the acquisition parameters: the repetition time is set to 1,500 milliseconds, the echo time is set to 135 milliseconds, and the total number of scans is determined to be 128 times - During the acquisition process, closely monitor the running status of the instrument and the quality of the acquired data. Ensure that the patient remains still during the scanning process to avoid the occurrence of artifacts and distortions in the images and spectral data due to body movement. If abnormal situations occur during the acquisition process, such as a sudden change in signal intensity or missing acquisition data, immediately stop the scanning, check the cause, and re-acquire. After the scanning is completed, conduct a preliminary inspection and storage of the acquired raw data for subsequent analysis 6. Data analysis and concentration quantification: - Use professional spectral analysis software to process the acquired spectral data. According to the preset algorithms and parameters, the software automatically identifies the peak of glutathione at 2.8 ppm in the proton spectrum and accurately calculates the area under the curve of this peak's waveform - Combine the calculated area under the curve data of the glutathione peak with the previously prepared external reference -the glutathione model. Through specific mathematical calculation methods and calibration formulas, quantify it into an absolute concentration value (mM). During the quantification process, factors such as the concentration of the external reference and the sensitivity of the instrument need to be considered, and necessary corrections and adjustments should be made to obtain accurate and reliable glutathione concentration detection results. Finally, review and record the quantified results to generate a detailed detection report

**Supplementary Table 2:** Social interaction test in animal experiments

Item	Description
Equipment	The experiment adopted a specially made plexiglass social interaction box. Its dimensions were 60 cm in length, 40 cm in width and 30 cm in height. The box was equally divided into three areas. The middle area served as a transition zone with a length of 20 cm and a width of 40 cm. The two side social areas were of the same size, both measuring 20 cm in length and 40 cm in width. In each social area, a small detachable metal wire mesh cage (with dimensions of 10 cm in length, 10 cm in width and 15 cm in height) could be placed, which was used to hold the experimental mice or the unfamiliar stimulus mice. The light intensity inside the box was maintained between 200 and 300 lux. The environmental temperature was kept stable within the range of 22 to 24 ^o^C, and the humidity was controlled between 40 and 60%. The whole experiment process was recorded by a high-definition camera installed on the top to ensure that the behavioral details of the mice could be clearly captured
Detailed process	Before the experiment began, the mice were placed in the experimental environment in advance to adapt for 1 to 2 hours. During the experiment, first, the test mice were gently placed into the middle transition area of the social interaction box and allowed to explore freely for 5 minutes. Subsequently, an unfamiliar mouse of the same sex, similar age, and without special treatment (the stimulus mouse) was put into the metal wire mesh cage in one of the social areas, while the metal wire mesh cage in the other social area was left empty. Then, the behavioral performance of the mice within 10 minutes was continuously observed and recorded, including the percentage of dwelling time in the social area where the unfamiliar mouse was present (Dwelling time), the dwelling frequency of entering that social area (Dwelling frequency), and the interaction count of direct interactions with the unfamiliar mouse (Interaction count)

**Supplementary Table 3:** ELISA kits used in animal experiments

Indicator	ELISA Kit
Glutathione	Glutathione Competitive ELISA Kit (EEL155, Thermo Fisher)
MDA	Malondialdehyde Competitive ELISA Kit (EEL160, Thermo Fisher)
8-OHdG	8-OHdG Competitive ELISA Kit (EEL004, Thermo Fisher)
PCD	Protein Carbonyl ELISA Kit (ab238536, Abcam)
IL-6	Mouse IL-6 ELISA Kit (BMS603-2, Thermo Fisher)
TNF-α	Mouse TNF alpha ELISA Kit (BMS607-3, Thermo Fisher)

MDA: malondialdehyde; 8-OHdG: 8-hydroxydeoxyguanosine, PCD: protein carbonyl derivatives; IL-6: interleukin 6; TNF-α: tumor necrosis factor-alpha.

**Supplementary Table 4:** Univariate relationship between glutathione levels and social phobia scores of subjects in the study

Variable	Model	OR (95%CI)	p-value
Peripheral glutathione			
LSAS score	Univariate	0.630 (0.446 - 0.890)	0.009
SADS score	Univariate	0.724 (0.519 - 1.011)	0.058
SAS score	Univariate	0.704 (0.496 - 0.998)	0.049
BSPS score	Univariate	0.731 (0.527 - 1.013)	0.060
Central glutathione			
LSAS score	Univariate	0.391 (0.265 - 0.577)	< 0.001
SADS score	Univariate	0.396 (0.270 - 0.579)	< 0.001
SAS score	Univariate	0.345 (0.231 - 0.515)	< 0.001
BSPS score	Univariate	0.473 (0.327 - 0.685)	< 0.001

Univariate logistic regression was adopted to assess the relationship between glutathione levels and social phobia scores. p < 0.05 suggested a statistically significant correlation.

OR: odds ratio; 95%CI: 95% confidence interval; LSAS: Liebowitz Social Anxiety Scale; SADS: Social Avoidance and Distress Scale; SAS: Social Anxiety Scale; BSPS: Brief Social Phobia Scale.
